# COVID-19, Coronavirus Vaccines, and Possible Association with Lipschütz Vulvar Ulcer: A Systematic Review

**DOI:** 10.1007/s12016-023-08961-5

**Published:** 2023-06-26

**Authors:** Stefano A. Vismara, Andrea Ridolfi, Pietro B. Faré, Mario G. Bianchetti, Sebastiano A. G. Lava, Samuele Renzi, Benedetta Terziroli Beretta Piccoli, Gregorio P. Milani, Lisa Kottanattu

**Affiliations:** 1 Department of Pediatrics, Kantonsspital Winterthur, Zurich, Switzerland; 2 Faculty of Biomedical Sciences, Università Della Svizzera Italiana, Lugano, Switzerland; 3 Pediatric Institute of Southern Switzerland, Ente Ospedaliero Cantonale, Bellinzona, Switzerland; 4 Clinic of Hematology, Oncology Institute of Southern Switzerland, Ente Ospedaliero Cantonale, Bellinzona, Switzerland; 5 Pediatric Cardiology Unit, Department of Pediatrics, Centre Hospitalier Universitaire Vaudois and University of Lausanne, Lausanne, Switzerland; 6 Heart Failure and Transplantation, Department of Pediatric Cardiology, Great Ormond Street Hospital, London, UK; 7 Division of Hematology and Oncology, CHUL-Laval, Quebec City, Canada; 8 Department of Pediatrics, Laval University, Quebec City, Canada; 9 Epatocentro Ticino, Lugano, Switzerland; 10 Faculty of Life Sciences & Medicine, King’s College London, King’s College Hospital, London, UK; 11 Pediatric Unit, Fondazione IRCCS Ca’ Granda Ospedale Maggiore Policlinico, Milan, Italy; 12 Department of Clinical Sciences and Community Health, Università Degli Studi Di Milano, Milan, Italy

**Keywords:** Acute genital ulcer, Coronavirus disease 2019, Immunization against coronavirus, Lipschütz ulcer

## Abstract

Lipschütz genital ulcer is a self-limited, non-sexually acquired disorder characterized by the sudden onset of a few ulcers. A primary Epstein-Barr virus infection is currently considered the most recognized cause. Recent reports document cases temporally related with coronavirus disease 2019 (COVID-19) or immunization against severe acute respiratory syndrome coronavirus 2 (SARS-CoV-2). We carried out a review of the literature to investigate the possible association between COVID-19 or the immunization against SARS-CoV-2 and genital ulcer. The pre-registered study (CRD42023376260) was undertaken following the Preferred Reporting Items for Systematic Reviews and Meta-Analyses methodology. Excerpta Medica, the National Library of Medicine, and Web of Sciences were searched. Inclusion criteria encompassed instances of acute Lipschütz ulcer episodes that were temporally linked to either COVID-19 or a vaccination against SARS-CoV-2. Eighteen articles were retained. They provided information on 33 patients 15 (14–24) years of age (median and interquartile range), who experienced a total of 39 episodes of Lipschütz ulcer temporally associated with COVID-19 (*N* = 18) or an immunization against SARS-CoV-2 (*N* = 21). The possible concomitant existence of an acute Epstein-Barr virus infection was excluded in 30 of the 39 episodes. The clinical presentation and the disease duration were similar in episodes temporally associated with COVID-19 and in those associated with an immunization against SARS-CoV-2. In conclusion, COVID-19 and immunization against SARS-CoV-2 add to Epstein-Barr virus as plausible triggers of Lipschütz genital ulcer.

**Supplementary Information:**

The online version contains supplementary material available at 10.1007/s12016-023-08961-5.

## Introduction

Vulvar ulcers are typically triggered by sexually transmitted germs such as Treponema pallidum, Herpes simplex virus, and, more rarely, some serovars of *Chlamydophila trachomatis*. Primary infection with human immunodeficiency virus may also present with vulvar ulcers. Noninfectious etiologies of vulvar ulcers are autoimmune conditions such as Crohn’s disease, Behçet’s disease, cancer (including leukemia), fixed drug eruption, and trauma [1, 2].

Lipschütz acute vulvar ulcer, first reported in Austria by B. Lipschütz (1878–1931) in 1912 as “ulcus vulvae acutum virginis” or “ulcus vulvae acutum pseudovenereum,” is a non-sexually transmitted condition. It usually occurs in adolescents and young adults and is characterized by a sudden onset of a few necrotic and painful genital ulcers. Restitutio ad integrum is the typical spontaneous course. Currently, available literature indicates that many cases of Lipschütz disease are associated with primary Epstein-Barr virus infection [1, 2].

Both coronavirus disease 2019 (COVID-19) and vaccines to prevent severe acute respiratory syndrome coronavirus 2 (SARS-CoV-2) infection have been temporally associated with Lipschütz ulcer [3]. However, single case reports cannot be employed to draw inferences about the relationships between COVID-19 or the immunization against SARS-CoV-2 and Lipschütz ulcer [3]. Hence, we performed a systematic review of the available literature on this issue.

## Methods

### Data Source

The study was recorded on the International Prospective Register of Systematic Reviews of the National Institute for Health Research (PROSPERO CRD42023376260) and undertaken in accordance with the Joanna Briggs Manual and the second edition [4] of the Preferred Reporting Items for Systematic Reviews and Meta-Analyses guidelines (PRISMA). Three databases, i.e., Excerpta Medica, the National Library of Medicine, and Web of Sciences, were explored in September 2022 and updated on January 15, 2023, without any restriction for original articles or letters using the following terms entered in separate pairs: “genital ulcer” OR “Lipschütz ulcer” OR “non-sexually acquired genital ulcer” OR “ulcus pseudovenereum” OR “ulcus vulvae (acutum)” AND “coronavirus disease 2019” OR “COVID 19” OR “SARS-CoV-2” OR “severe acute respiratory syndrome coronavirus 2.” The search strategy for each database is given in the online supplementary material. Google Scholar, personal files, and the bibliography of each identified article were also screened. Cases published uniquely as abstracts were not included. 

#### Selection Criteria—Diagnostic Criteria

Of interest were acute episodes of Lipschütz ulcer temporally associated with a COVID-19 or a vaccination against SARS-CoV-2 and a latency of four weeks or less [5]. The Lipschütz ulcer was categorized as intra-infectious in cases exhibiting both COVID-19 and genital disease simultaneously. On the other hand, it was categorized as postinfectious in cases with the onset of the genital disease after recovery from COVID-19. Cases of Lipschütz ulcer in patients without any symptom of COVID-19 but uniquely with a positive test were considered intra-infectious. In the case of post-vaccination ulcer, the time latency from vaccination against SARS-CoV-2 to the onset of Lipschütz ulcer was calculated. Cases heralded by an infection other than COVID-19 or by a vaccination against a microorganism other than SARS-CoV-2 with a latency of four weeks or less were not included. The diagnosis was made in previously healthy and apparently immunocompetent subjects with rapid onset of a few, painful, rather large genital ulcers. Sexual inactivity was not a prerequisite for the diagnosis [1, 2]. Patients with a sexually transmitted disease, laboratory findings consistent with an acute Epstein-Barr virus infection, Behçet’s disease, Crohn’s disease, cancer, cutaneous drug reactions, or trauma were excluded.

A positive microbiologic testing was a sine qua non for the diagnosis of COVID-19. Its severity was classified into five stages, as suggested by the National Institutes of Health [5]: (1) asymptomatic (without symptoms and signs), (2) mild (any symptom or sign such as malaise, headache, fever, cough, sore throat, or myalgia without shortness of breath, dyspnea, or abnormal chest imaging), (3) moderate (evidence of lower respiratory tract disease by clinical assessment or imaging, and oxygen saturation on room air ≥ 94%), (4) severe (respiratory rate > 30/min, oxygen saturation on room air < 94%, or pulmonary infiltrates > 50%), and (5) critical (hypercapnia, septic shock, or multiple organ dysfunction).

### Data Extraction—Reporting Comprehensiveness—Data Synthesis

Data were extracted using a piloted form. For each episode of Lipschütz ulcer temporally related to COVID-19 or an immunization against SARS-CoV-2, the following information was sorted: demographics; medical history; temporal relationship between coronavirus disease 2019 or immunization against it and first symptoms and signs of genital ulcer; clinical features of Lipschütz ulcer such as local clinical features, urinary symptoms, treatment with systemic corticosteroids, and disease duration (defined as recovery time of ulcers); and the possible existence of an acute Epstein-Barr virus serology test.

For each episode of Lipschütz ulcer temporally related to COVID-19, information about microbiology testing and features of coronavirus disease was also collected. For each episode of Lipschütz ulcer heralded by a vaccination against SARS-CoV-2, information about the type of vaccine and dose (first, second, or third) was collected. The corresponding authors of original reports were contacted to obtain missing information.

Reporting comprehensiveness was stratified as satisfactory, good, or excellent according to our standard procedure [2]. 

Two authors separately performed the literature search, the selection of eligible studies, data extraction, and evaluation of reporting comprehensiveness in duplicate. Any disagreements were discussed, and a senior author was involved for any remaining discrepancies. 

Pairwise deletion was employed to deal with missing values. Continuous variables are shown as median with interquartile range and were analyzed using the Wilcoxon-Mann–Whitney test. Categorical variables are shown as proportions. Dichotomous categorical data were compared using the Fisher test, and ordered categorical data were compared using the Wilcoxon-Mann–Whitney test. A two-sided *P* < 0.05 was used to denote significance. GraphPad Prism 9.5.1 (GraphPad Software, San Diego, California, USA) was used for statistics.

## Results

### Search Results

**Fig. 1:**
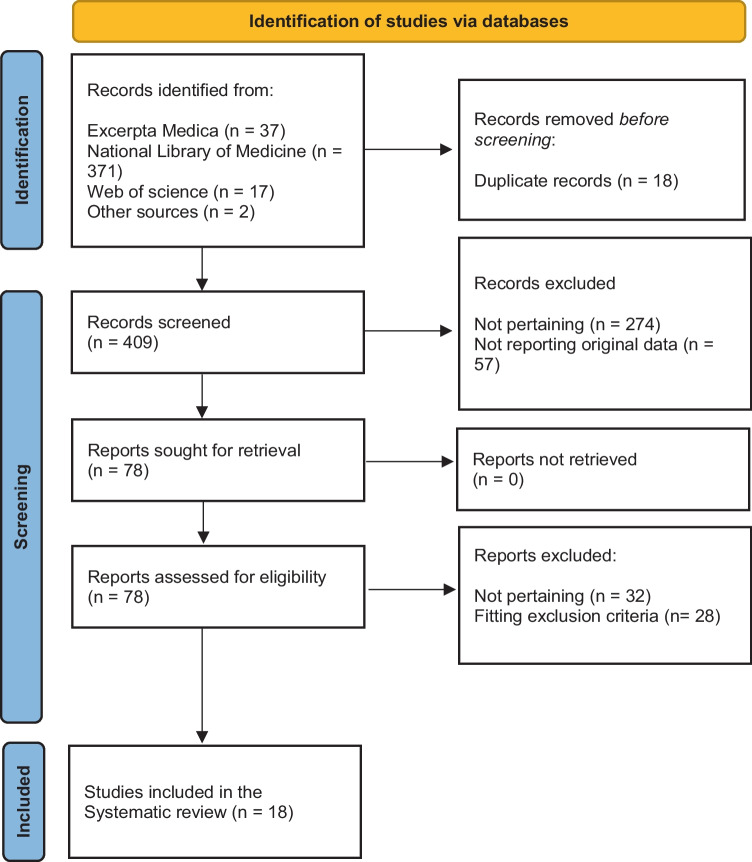
Acute Lipschütz vulvar ulcer temporally associated with coronavirus disease 2019 or immunization against coronavirus. Flowchart of the literature search process

For the final analysis, we retained 18 communications [6–23] published between 2020 and 2023 in English (*N* = 15), Spanish (*N* = 2), or Italian (*N* = 1). Flowchart of study selection is shown in Fig. 1. They had been reported from the following continents: 10 from America (USA, *N* = 9; Brazil, *N* = 1); six from Europe (Spain, *N* = 3; Italy, *N* = 1; Portugal, *N* = 1; Switzerland, *N* = 1), and two from Oceania (Australia, *N* = 2). The reports described 33 previously healthy subjects 15 (14–24) years of age, who experienced 39 episodes of Lipschütz vulvar ulcer. Reporting comprehensiveness was excellent in 24, good in 11, and satisfactory in four episodes of acute genital ulcer (Supplementary Tables 1 and 2).

### Findings

The characteristics of the 33 patients with 39 episodes of Lipschütz ulcer are given in Table 1. Eighteen episodes were temporally associated with COVID-19 and 21 with an immunization against SARS-CoV-2. All episodes temporally associated with COVID-19 were intra-infectious.

**Table 1:** Clinical features of 33 patients 15 (14–24) years of age with 39 acute episodes of acute Lipschütz vulvar ulceration temporally associated with coronavirus disease 2019 (COVID-19) or immunization against severe acute respiratory syndrome coronavirus 2 (SARS-CoV-2). Five patients also presented a further episode of acute Lipschütz vulvar ulceration temporally associated with a flu-like disease

	**Total**	**COVID-19**^a^	**Immunization against SARS-CoV-2**	***P*-value**
Episodes, *N*	39	18	21^b^	
Local presentation				
Mirror-like appearance	13	5	8	0.734
Number of ulcers				0.124
1, *N*	9	8	1	
2, *N*	7	2	5	
3, *N*	6	3	3	
≥ 4, *N*	6	3	3	
No information, *N*	11	2	9	
Voiding disorders				
Dysuria alone, *N*	13	6	7	0.999
Urinary retention, *N*	3	1	2	0.999
Inguinal adenopathy*, N	4	2	2	0.999
Disease duration				
Days	14 (7–19)	14 (7–15)	14 (7–21)	0.685
≥ 28 days	4	2	2	0.999
Systemic corticoids, *N*	9	4	5	0.999

^a^All cases exhibited vulvar ulcer during COVID-19

^b^Comirnaty (BNT162b2), *N* = 11; Vaxzevria (ChAdOx1-S), *N* = 6; Spikevax (mRNA-1273), *N* = 4

^*^Palpable inguinal lymph nodes

The laboratory method used for the diagnosis of COVID-19 was serology certified for the diagnosis of an acute coronavirus infection in one case, antigen test in one, and polymerase chain reaction in 15 cases (this information was not available for one case). COVID-19 was mild in 17 and moderate in one case. Most immunization-associated cases were observed after Comirnaty (BNT162b2). Four episodes occurred after the first, nine after the second, and three after the third vaccination (this information was not available for the remaining episodes). The time latency between the vaccination and the onset of Lipschütz ulcer was 4 days or less for all but one case.

Twenty-nine patients experienced one episode of Lipschütz ulcer, three patients two episodes [11, 16, 23], and one patient four episodes [11]. Four patients with two or more episodes experienced both infection- and immunization-associated attacks of Lipschütz ulcer. The patient with four episodes had a first attack associated with a mild form of COVID-19 and three attacks after immunization against SARS-CoV-2. The first two episodes occurred after Spikevax (mRNA-1273) and the third after Comirnaty (BNT162b2).

Three subjects developed the characteristic features of Lipschütz ulcer both after a Comirnaty (BNT162b2) vaccination and in the context of a mild COVID-19. Interestingly, these three subjects had a history of Lipschütz ulcer associated with a flu-like disease [11, 19, 23]. Two further patients, an 11-year-old and a 15-year-old girl, had a recurrence of Lipschütz ulcer associated with a flu-like disease and an Influenza A infection 7 and 3 months later, respectively [23].

Most episodes presented with two to four painful vulvar ulcers, often with a mirror-like appearance. An inguinal adenopathy was documented only in slightly more than 10% of cases. Dysuria, occasionally associated with urinary retention, occurred in every second case. Systemic corticosteroids were prescribed in approximately every fourth case. The disease remitted on average after 14 days.

The clinical presentation, the management, and the disease duration were similar in episodes temporally associated with COVID-19 and in those associated with an immunization against SARS-CoV-2.

The possible concomitant existence of an acute Epstein-Barr virus infection was excluded serologically in 30 of the 39 episodes (infection-associated episodes, *N* = 15; vaccination-associated episodes, *N* = 15).

## Discussion

The present systematic literature review documents 39 episodes of Lipschütz vulvar ulcer temporally associated with COVID-19 or, more frequently, an immunization against SARS-CoV-2.

A temporal relationship between COVID-19 or vaccines to prevent SARS-CoV-2 infection does not immediately imply causality. Nonetheless, three factors insinuate that the link between COVID-19 or immunization against SARS-CoV-2 and Lipschütz ulcer may be causal. First. Primary Epstein-Barr virus infection, which is currently regarded as the most characteristic cause of genital ulcer, was excluded in most cases. Furthermore, approximately 40 patients with Epstein-Barr virus-associated Lipschütz ulcer were published over a period of 50 years, while 33 patients are documented in the present review that covers just 3 years of coronavirus pandemic. Second. Approximately 10% of patients included in this review experienced recurrences after re-exposure to coronavirus 2 or vaccination against it. Third, a cause-and-effect relationship between coronavirus 2 and Lipschütz ulcer appears plausible also because ulcerations of the oral mucosa are detected in two-thirds of individuals with coronavirus disease 2019 [24]. Moreover, genital ulcers were reported also in two male subjects affected by coronavirus disease 2019 [25, 26]. SARS-CoV-2 has been observed to elevate levels of cytokines, including tumor necrosis factor α, which can impact the adhesion of endothelial cells and chemotaxis of neutrophils, ultimately resulting in the development of aphthous lesions [13]. However, the pathophysiology of ulcers affecting the vulvar region remains uncertain [3].

The clinical features of Lipschütz ulcer associated with COVID-19 or immunization against SARS-CoV-2 and that of the remaining cases, including those associated with Epstein-Barr virus, are undistinguishable. Remarkably, while approximately 10% of cases associated with COVID-19 or vaccination against SARS-CoV-2 recur, such a propensity has been very rarely (about 1%) observed before the pandemic. This likely relates to the fact that diseases such as Epstein-Barr virus mononucleosis can only be caught once [27]. However, COVID-19 may recur. Furthermore, vaccines are widely prescribed to prevent and control the pandemic.

Most cases of Lipschütz ulcer included in this analysis were induced by a vaccine against SARS-CoV-2. Interestingly, Lipschütz ulcer has never been reported to be temporally associated with other vaccines.

Since Lipschütz ulcer spontaneously resolves, the management is essentially supportive and includes reassurance, local care, and pain control. Patients and caregivers should be reassured that the disorder is not sexually transmitted and that recurrence is possible, but unusual [1, 2]. Systemic corticosteroids are often administered but do not reduce disease duration [2].

This study has limitations. First, it included a uniquely small number of individually documented cases. Furthermore, available literature data do not allow any formal meta-analysis. Third, being a systematic review of individual cases, a formal assessment of the risk of bias was not possible.

In conclusion, the present systematic literature review indicates that COVID-19 and immunization against SARS-CoV-2 add to Epstein-Barr virus as plausible triggers of Lipschütz vulvar ulcer. Medical practitioners should give due consideration to the possibility of the aforementioned two conditions in individuals presenting with vulvar ulcer. Finally, forthcoming investigations pertaining to novel coronavirus vaccines ought to examine the incidence of vulvar ulcer in the female population.

### Supplementary Information

Below is the link to the electronic supplementary material.

Supplementary file1 (DOCX 32 KB)

## Data Availability

The data supporting this study are available from the corresponding author upon reasonable request.
