# Supplementary material for: COVID-19, Coronavirus Vaccines, and Possible Association with Lipschütz Vulvar Ulcer: A Systematic Review
Source: Clin Rev Allergy Immunol. 2023 Jun 26;65(2):166–71. doi: 10.1007/s12016-023-08961-5 (PMC10567961; doi:10.1007/s12016-023-08961-5)
Supplement: Supplementary file 1 — Supplementary file1 (DOCX 32 KB) [file 12016_2023_8961_MOESM1_ESM.docx]

**Supplementary online material**

**COVID-19, coronavirus vaccines and possible association with Lipschütz vulvar ulcer: systematic review**

**Literature search strategy**

**Excerpta Medica**

#1'coronavirus disease 2019' OR (covid AND 19) OR 'sars cov 2' OR (severe AND acute AND respiratory AND syndrome AND coronavirus AND 2); #2'genital ulcer' OR 'lipschütz ulcer' OR 'non-sexually acquired genital ulceration' OR 'ulcus pseudovenereum' OR 'ulcus vulvae (acutum)'

Final string = #1 AND #2

**National Library of Medicine**

((((coronavirus disease 2019 [All Fields]) OR (COVID 19[All Fields])) OR (SARS-CoV-2[All Fields])) OR (severe acute respiratory syndrome coronavirus 2[All Fields])) AND ((((((genital ulcer[All Fields]) ) OR (Lipschütz ulcer[All Fields])) OR (non-sexually acquired genital ulceration[All Fields])) OR (ulcus pseudovenereum[All Fields])) OR (ulcus vulvae (acutum)[All Fields]))

**Web of Science**

#1 genital ulcer (All Fields) or Lipschütz ulcer (All Fields) or ulcus pseudovenereum (All Fields) or ulcus vulvae (acutum) (All Fields) #2 coronavirus disease 2019 (All Fields) or COVID 19 (All Fields) or SARS-CoV-2 (All Fields) or severe acute respiratory syndrome coronavirus 2 (All Fields)

Final string = #2 AND #

**Supplementary table 1**: Episodes of Lipschütz ulcer associated to COVID-19 and report completeness. All vulvar ulcers developed during COVID-19.

| **Autor** | **Year** | **Age subject**  **(years)** | **Number of ulcers** | **Disease duration**  **(days)** | **Technique for Covid-19 diagnosis** | **EBV Infection Excluded** | **Report completeness** |
| --- | --- | --- | --- | --- | --- | --- | --- |
| Christl J. [7] | 2021 | 41 | 2 | 14 | PCR | Yes | Excellent |
| Falkenhain-Lopez D. [9] | 2020 | 19 | 1 | 14 | PCR | No | Excellent |
| Hsu T. [11] | 2022 | 14 | 4 | 28 | Not specified | Yes | Good |
| Hsu T. [11] | 2022 | 29 | 2 |  | Antigenic Test | No | Good |
| Jacyntho CM. [12] | 2022 | 35 | 6 | 7 | PCR | Yes | Excellent |
| Krapf JM. [13] | 2021 | 13 |  | 40 | PCR | Yes | Excellent |
| Rubin ES. [18] | 2020 | 26 | 1 | 7 | PCR | No | Good |
| Rodríguez Blanco H. [17] | 2021 | 14 | 1 | 21 | PCR | Yes | Excellent |
| Ridolfi A. [16] | 2022 | 15 | 3 | 14 | PCR | Yes | Excellent |
| Alberelli MC. [6] | 2021 | 10 | 3 | 14 | Serology | Yes | Excellent |
| Morais ML. [14] | 2022 | 17 | 3 | 14 | PCR | Yes | Good |
| Schmitt T. [20] | 2022 | 15 | 5 | 14 | PCR | Yes | Excellent |
| Crofts VL. [23] | 2022 | 15 | 1 |  | PCR | Yes | Good |
| Crofts VL. [23] | 2022 | 14 | 1 | 14 | PCR | Yes | Excellent |
| Crofts VL. [23] | 2022 | 15 | 1 | 7 | PCR | Yes | Excellent |
| Crofts VL. [23] | 2022 | 12 |  |  | PCR | Yes | Good |
| Crofts VL. [23] | 2022 | 11 | 1 | 5 | PCR | Yes | Excellent |
| Crofts VL. [23] | 2022 | 15 | 1 | 10 | PCR | Yes | Excellent |

* Information was asked to the corresponding author of the paper

**Supplementary table 2**: Episodes of Lipschütz ulcer temporally associated to a vaccination against severe acute respiratory syndrome coronavirus 2 and report completeness.

| **Author** | **Year** | **Age of the subject**  **(years)** | **Number of ulcers** | **Disease duration**  **(days)** | **Vaccine** | **Latency after vaccine**  **(days)** | **Report completeness** |
| --- | --- | --- | --- | --- | --- | --- | --- |
| Drucker A. [8] | 2022 | 14 | 3 | 10 | Comirnaty | 2 | Excellent |
| González-Romero N.[19] | 2021 | 24 | 4 | 21 | Vaxzevria | 3 | Excellent |
| González-Romero N.[19] | 2021 | 18 |  |  | Vaxzevria | 1 | Satisfactory |
| González-Romero N.[19] | 2021 | 25 |  |  | Vaxzevria | 2 | Satisfactory |
| González-Romero N.[19] | 2021 | 24 |  |  | Vaxzevria | 1 | Satisfactory |
| González-Romero N.[19] | 2021 | 25 |  |  | Vaxzevria | 2 | Satisfactory |
| Hsu T. [11] | 2022 | 12 |  | 14 | Comirnaty | 2 | Excellent |
| Hsu T. [11] | 2022 | 14 | 4 | 28 | Comirnaty | 3 | Excellent |
| Hsu T. [11] | 2022 | 29 | 2 |  | Spikevax | 1 | Excellent |
| Hsu T. [11] | 2022 | 29 | 2 |  | Spikevax | 2 | Excellent |
| Hsu T. [11] | 2022 | 29 | 2 |  | Spikevax |  | Excellent |
| Popatia S. [15] | 2022 | 12 | 3 | 10 | Comirnaty | 3 | Excellent |
| Wijaya M. [21] | 2022 | 16 | 4 | 28 | Comirnaty | 3 | Excellent |
| Wijaya M. [21] | 2022 | 14 | 2 | 7 | Comirnaty | 4 | Excellent |
| Wijaya M. [21] | 2022 | 19 |  | 7 | Vaxzevria | 3 | Good |
| Wojcicki AV. [22] | 2022 | 16 | 1 | 20 | Comirnaty | 1 | Excellent |
| Ridolfi A. [16] | 2022 | 15 | 3 | 14 | Comirnaty | 4 | Excellent |
| Salusti-Simpson M. [19] | 2022 | 22 | 2 |  | Comirnaty | 2 | Good |
| Crofts VL. [23] | 2022 | 17 |  | 5 | Comirnaty | few days | Good |
| Crofts VL. [23] | 2022 | 12 |  | 10 | Spikevax | 3 | Good |
| Crofts VL. [23] | 2022 | 15 |  | 6 | Comirnaty | 1 | Good |
